# From “shooting in the dark” to “precision hit”: Technological evolution, synergistic strategies, and future paths for peripheral pulmonary nodule diagnosis

**DOI:** 10.1016/j.pccm.2026.03.001

**Published:** 2026-06-06

**Authors:** Ximing Liao, Qiang Li, Ko-pen Wang

**Affiliations:** a Department of Pulmonary and Critical Care Medicine, Shanghai East Hospital, School of Medicine, Tongji University, Shanghai 200120, China; b Department of Pulmonary and Critical Care Medicine, Johns Hopkins University School of Medicine, Baltimore, MD 21224, USA

Peripheral pulmonary nodules (PPNs) are pivotal for early lung cancer screening, yet their transbronchial diagnosis has long posed a dilemma: like “shooting a moving target with eyes closed”. This “blindness”, rooted in the inability to visualize lesions in real time and adapt to respiratory motion, has driven decades of technological evolution, from manual bronchoscopes to robotic systems, and from X-ray fluoroscopy to multimodal navigation. Notably, each era of technological innovation has aimed to address phase-specific problems inherited from the previous stage, yet none has broken through the core limitation of “continuous real-time verification throughout the diagnostic process”, leading to the persistent “blindness” dilemma even with cumulative progress.

Amid this progress, one technology remains irreplaceable: Transbronchial needle aspiration (TBNA), which bridges the gap between “locating lesions” and “obtaining diagnostic samples”. This editorial offers a concise review of the evolutionary journey of PPN diagnosis, dissects why such “blindness” persists, and maps a path forward—all while inviting fellow pulmonologists to reflect on a potential yet easily overlooked direction that fulfills the core goal of enhancing accuracy without compromising safety or practicality.

## Evolution of PPN diagnosis: from “blind sampling” to “guided precision”

**Fig. 1:**
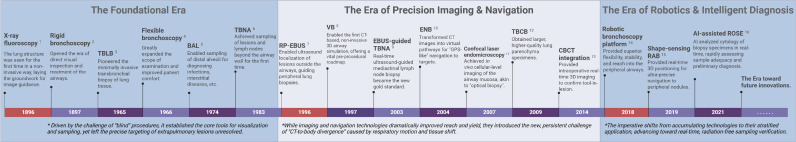
Timeline of bronchoscopic diagnostic technological advancements for peripheral pulmonary nodules. The timeline delineates three evolutionary eras, mapping key technological innovations, their clinical contributions, and persistent challenges in PPN diagnosis, with a forward-looking perspective on future developments. AI, Artificial intelligence; BAL, Bronchoalveolar lavage; CBCT, Cone-beam computed tomography; CT, Computed tomography; EBUS, Endobronchial ultrasound; ENB, Electromagnetic navigation bronchoscopy; GPS, Global positioning system; PPNs, Peripheral pulmonary nodules; RAB, Robotic-assisted bronchoscopy; RP-EBUS, Radial probe endobronchial ultrasound; ROSE, Rapid on-site evaluation; TBCB, Transbronchial cryobiopsy; TBLB, Transbronchial pulmonary biopsy; TBNA, Transbronchial needle aspiration; VB, Virtual bronchoscopy.

The success of PPN diagnosis relies on five sequential key steps, which serve as the core goal driving technological progress. These steps are: visualize the lesion; clarify the relationship between the lesion and the airway; reach the vicinity of the lesion; access the lesion; and collect specimens under direct visualization. Against this backdrop, the journey to overcome “blindness” in PPN diagnosis unfolds in three defining phases, each striving to align technology with these core steps (Fig. 1).^1^, ^2^, ^3^, ^4^, ^5^, ^6^, ^7^, ^8^, ^9^, ^10^, ^11^, ^12^, ^13^, ^14^, ^15^, ^16^

*The foundational era*: Early PPN diagnosis relied on manual bronchoscopes and chest X-rays, leading to high false-negative rates due to limited visualization. A breakthrough came with the refinement of TBNA, paired with C-arm fluoroscopy. This combination provided “real-time on-site guidance”, allowing clinicians to align the needle tip with nodule shadows across multiple imaging planes, verify positioning during respiration, and sample extrapulmonary lesions by piercing the airway wall. This approach addressed the “reach problem” of traditional bronchoscopes, making TBNA a routine standard procedure—laying the groundwork for all subsequent advances. While this approach, which relied on two-dimensional shadows, addressed the reach problem, it merely transformed absolute blindness into targeting ambiguity, a fundamental limitation that subsequent eras of precision imaging would strive to overcome.

*The era of precision imaging & navigation*: Radial probe endobronchial ultrasound (RP-EBUS), a real-time imaging confirmation tool alongside fluoroscopy and cone-beam computed tomography (CBCT), was developed early to clarify extrapulmonary lesions and address “blindness” by verifying lesion location relative to airways. Although RP-EBUS can be integrated with multiple navigation tools, tissue sampling remains “blind” without real-time verification of tissue acquisition. The advent of computed tomography (CT) spurred two key innovations: Virtual bronchoscopy (VB) and electromagnetic navigation bronchoscopy (ENB). VB created preoperative “airway maps” to plan paths, while ENB reduced X-ray reliance by using electromagnetic fields for localization. Yet both had critical flaws: VB was static (like an “old map”), failing to adapt to respiratory motion, while ENB risked “chasing false lesions” if anatomy shifted. Throughout this era, TBNA remained central: navigation systems only guided the needle to the lesion vicinity—TBNA was still needed to puncture and sample, turning “path planning” into actionable results. Notably, some perspectives hold that TBNA yields a low diagnostic yield for ground-glass opacities; thus, transbronchial cryobiopsy (TBCB) was developed during this period as a complementary approach. Although TBCB is unable to directly access extrapulmonary lesions, it can be combined with existing techniques via tunneling to achieve synergistic diagnostic efficacy. Thus, this era did not bring an end to “blindness”, but shifted its locus: it replaced the ambiguity of where to shoot with the precision of how to reach the target. This, in turn, exposed a more insidious gap, namely the chasm between flawless procedural navigation and the ongoing uncertainty of diagnostic confirmation, thereby defining the exact frontier for the next technological leap.

*The era of robotics & intelligent diagnosis*: Robotic bronchoscopes marked a leap in “reach, control, and stability”, with two dominant designs: one uses a reinforced outer sheath to deliver TBNA needles to distal bronchi while maintaining a stable catheter angle, and the other leverages shape-sensing technology to dynamically align the needle with lesions and lock it into place. Beyond reducing physician fatigue and improving angular precision, a key advantage of robotic systems lies in their ability to maintain consistent catheter alignment with the lesion—offsetting minor shifts from respiratory motion or operator tremor, and thus laying a solid foundation for precise TBNA puncture. In parallel, artificial intelligence further advances robotic platforms by enabling automated nodule detection, preoperative path planning, and real-time needle-lesion alignment feedback. When combined with artificial intelligence-supported rapid on-site evaluation for efficient, objective assessment of sample adequacy, this intelligent assistance further reduces procedural blindness and supports consistent, reliable TBNA performance. Yet even with these advances, robotic and intelligent tools do not replace TBNA; instead, they enhance its reliability in complex scenarios. For example, robotic systems excel at delivering TBNA needles to tiny, deep-seated nodules that are often missed by manual techniques, and then verify needle placement with C-arm fluoroscopy or CBCT. But here’s the catch: no matter how advanced these robotic systems are, they’re still essentially “high-tech assistants” that can’t act on autonomously, and they can be thought of as “souped-up cars” tailored for the lung’s “narrow alleys”. In essence, robotic bronchoscopes are still human-controlled machines. They still require human judgment and technical skill to operate. To use a car analogy: The “car body” is smaller, allowing it to enter narrower “alleys” (smaller bronchi). The accelerator is replaced by a lever for forward/backward movement. The steering wheel is replaced by a trackball. It can be operated without physical proximity to the device, but it still requires mastering basic “driving skills”, relying on human judgment and real-time checks to enhance diagnostic reliability in complex cases. Therefore, the robotic era represents the culmination of the guidance quest, mastering the lung’s anatomy with engineered precision. Yet, by achieving near-perfect deliverability, it starkly isolates the final, unaddressed core issue regarding “blindness”. This engineering triumph also compels a reflection: by stacking advanced technologies primarily to ensure reach, have we constructed an overly complex solution that, for many nodules, may exceed the clinically necessary threshold?

## Current pain points: why “shooting in the dark” persists

As detailed in the technological evolution above, decades of technical progress have only resolved some diagnostic challenges but failed to eradicate the core blindness dilemma. The persistence of this dilemma stems from four interconnected, unresolved gaps—the cumulative manifestation of inherent technological limitations across eras.

*CT-to-body discrepancy—static planning vs. dynamic anatomy*: All modern navigation technologies, tunnel techniques (e.g., LungPro), and robotic bronchoscopes, share a common flaw: the mismatch between preoperative CT imaging and real-time anatomical changes. Respiratory motion, tissue deformation, or patient movement can cause lesions to deviate from their predicted positions, leaving clinicians reliant on outdated “maps”. Even CBCT, which confirms needle placement, only provides a “snapshot” and cannot guide the puncture process itself. Consequently, as a direct countermeasure to this core challenge, sophisticated anesthesia and ventilation strategies have become an indispensable component of modern biopsy protocols. Clinical practice employs optimized ventilation parameters,^17^ apneic techniques,^18^ and even positional adjustments^19^ to compensate for respiratory motion, aiming to bridge the gap between preoperative planning and intraoperative anatomy, thereby providing navigation with a relatively stable “real-target” environment. However, this complex, multidisciplinary approach also reveals itself as a transitional workaround: in the absence of real-time, continuous target visualization, we compensate by stacking procedural complexity to achieve targeting reliability.

*Trade-offs in non-X-ray alternatives: radiation reduction vs. functional limitations*: Non-X-ray modalities, such as ultrasound, magnetic resonance imaging (MRI), and optical coherence tomography (OCT), eliminate radiation exposure but suffer inherent limitations: Ultrasound is blocked by lung air, making deep nodules invisible; MRI is expensive and incompatible with metal implants, restricting its use; OCT has limited penetration depth, failing to visualize nodule boundaries in peripheral areas. While these tools act as “extra eyes” for surface lesions, they cannot replace real-time motion tracking (like fluoroscopy), leaving no way to dynamically adjust to nodule movement and resulting in “blind” punctures.

*Lack of head-to-head validation: innovation hype vs. proven efficacy*: Emerging tools (robotic bronchoscopes, magnetic navigation) are often evaluated in isolation, not directly against established techniques like fluoroscopy-guided TBNA. Most studies only compare new technologies to literature-reported data on traditional methods, rather than setting rigorous direct control groups; this leads to publication bias and selection bias, misjudging new tools’ actual targeting ability and fostering undue confidence. Without high-quality randomized controlled trials (RCTs) to quantify their marginal benefits over conventional bronchoscopy (e.g., whether they truly improve hit rates), clinicians may choose unproven new tools over traditional methods that already enable accurate targeting—exacerbating “blindness” due to misselection.

*Risk of “overkill”: technological sophistication vs. targeted application*: Robotic bronchoscopes undoubtedly excel at addressing the core pain point of “failing to effectively hit nodules directly” in complex scenarios. Their precise maneuverability and ability to reach hard-to-access lesions make up for traditional techniques’ limitations, which often struggle to target these complex nodules, and boost accurate hit rates, marking a key advancement in PPN diagnosis. Yet for simpler cases, like near-chest wall nodules where established methods (transthoracic needle aspiration [TTNA] or fluoroscopy-guided TBNA) already reliably achieve direct hits and avoid “blindness”, thoughtful discussion is warranted. This is not intended to undermine their value in solving “missed hits”, but rather to question whether the universal application—even in cases that are well-managed by traditional techniques—aligns with optimal clinical practice and resource allocation, and leverages their strengths for truly complex scenarios.

Taken together, these limitations indicate that “blindness” is not a transient technical flaw, but a structural constraint of transbronchial diagnosis. Any future diagnostic system that fails to provide continuous, real-time confirmation throughout the sampling process, rather than intermittent verification, will inevitably reproduce the same failure modes, regardless of how advanced individual components appear.

## Solutions anchored in “TBNA + multimodal synergy”

To break this deadlock, the potential key lies not in pursuing standalone “revolutionary” technologies, but in building TBNA-based multi-modal strategies paired with clinical stratification that may directly address the aforementioned gaps.

*TBNA as the “core executor”*: TBNA is the backbone of PPN diagnosis, with value that transcends technological trends. It breaks airway barriers by puncturing the airway wall to reach extrapulmonary lesions—a capability no navigation system can achieve alone. For narrow spaces where bronchoscopes or catheters cannot access, the thinner and more flexible “micro-TBNA” acts as a “small pistol”, delivering precise shoots (punctures) to obtain tissue sample. Most critically, TBNA establishes a connection between guidance and diagnosis: Whether paired with fluoroscopy, ENB, or robots, it transforms “path planning” into usable diagnostic specimens—without it, navigation remains a “map without a destination”.

*Combining strengths to reduce blindness by multimodal synergy*: To address the core pain point of “failing to effectively hit nodules directly”, the optimal strategy relies on integrating complementary tools to offset the flaws of individual technologies. For example, the combination of fluoroscopy, TBNA, and RP-EBUS leverages the strengths of each modality: fluoroscopy enables real-time tracking of nodule motion, which is critical for compensating respiratory motion; RP-EBUS confirms the needle’s proximity to the lesion, eliminating uncertainty about target location; and TBNA enables precise sampling. The multimodal synergy balances safety (minimizing the risk of pneumothorax) and accuracy, building on fluoroscopy’s real-time advantage while incorporating intrabronchial visual verification to avoid blind puncture. Similarly, the integration of ENB, TBNA, and CBCT addresses the “chasing shadows” issue associated with standalone navigation: ENB uses electromagnetic guidance to direct TBNA to the lesion’s vicinity (solving the “where to go” problem), while CBCT verifies the needle’s actual placement against real-time anatomy (correcting discrepancies between preoperative CT and in-body anatomical changes), ensuring that TBNA sampling is grounded in physical reality rather than outdated imaging maps, and thereby further reducing the risk of missed targets. It is noteworthy that the key principle must be emphasized: assembling the “diagnostic weapon” with the most essential components.

*Clinical stratification: avoiding overuse with a “diagnostic ladder”*: To ensure practicality and cost-effectiveness, a stepwise approach is critical (Supplementary Fig. 1): (1) Low-risk nodules: Use low-dose CT for follow-up—no invasive testing needed. (2) Simple cases: For near-chest wall or larger nodules, use TTNA or fluoroscopy-guided TBNA—proven, low-cost, and sufficient. (3) Complex cases: Reserve robotic bronchoscopes for tiny, deep-seated nodules or patients with poor lung function—cases in which simpler methods fail to balance safety and accuracy.

## Future outlook

A novel medical technology can gain a firm foothold in clinical practice only when it meets five core criteria: firstly, it must guarantee accuracy and safety; secondly, it should be simple and easy to operate; thirdly, it ought to offer favorable cost-effectiveness; fourthly, it should exhibit clear superiority over existing methods; and fifthly, it should address broad clinical demands. These criteria provide a practical benchmark against which future developments in PPN diagnosis should be judged, particularly in efforts to overcome persistent diagnostic “blindness”.

In the near term, the most pressing priority is not the continued proliferation of new navigation platforms, but rigorous head-to-head validation of TBNA-centered multimodal strategies against established fluoroscopy-guided TBNA. Without such comparative evidence, further technological stacking poses risks of amplifying procedural complexity and resource utilization without achieving proportional gains in diagnostic yield. At this stage, prioritizing well-designed comparative studies, standardized outcome measures, and structured operator training is more likely to improve diagnostic performance than incremental device innovation alone.

Over the mid-term, development should focus on transforming lesion confirmation from an intermittent checkpoint into a continuous process integrated throughout sampling. The persistence of diagnostic “blindness” reflects not a failure of reach, but the absence of real-time feedback during needle advancement and tissue acquisition. Artificial intelligence may serve as an enabling layer by fusing preoperative CT data with real-time respiratory signals to model lesion motion trajectories. When combined with the ability of robotic bronchoscopic platforms to maintain stable catheter orientation and fixed puncture angles, such integration may allow dynamic adjustment of TBNA trajectories and partially mitigate respiratory-induced target displacement, thereby shifting transbronchial biopsy toward a more adaptive and responsive workflow.

In the longer term, the pursuit of radiation-free real-time guidance should be framed as a strategic goal rather than a purely technological aspiration. Research should prioritize advancing non-X-ray imaging modalities, including air-resistant ultrasound probes capable of visualizing deep pulmonary nodules and low-cost MRI systems compatible with interventional and robotic environments. The objective is not simply to replace fluoroscopy, but to preserve TBNA’s sampling efficacy while reducing cumulative radiation exposure for both patients and clinicians. At the same time, continued emphasis on head-to-head evaluation of emerging tools against established TBNA-based approaches remains essential, as even the most advanced platforms ultimately depend on clinicians’ ability to interpret anatomy, judge puncture trajectories, and execute sampling effectively. Moreover, we must constantly reflect on whether our PPN diagnostic path has deviated or even regressed, guarding against compounding errors such as false goals and wasteful overinvestment in target localization. Ultimately, we must strive to identify a truly real-time, simpler, and more accurate approach.

The journey to diagnose PPNs has come a long way from “blind bronchoscopy”, but “shooting in the dark” persists—not for lack of technology, but for lack of synergy. TBNA, often overshadowed by newer tools, is the unifying thread: it connects traditional fluoroscopy to modern robots, and navigation systems to actionable diagnoses. The future lies not in chasing “over-elaborate” standalone tools, but in “TBNA-centered multimodal strategies” that are powered by new diagnostic technologies, stratified according to need, and based on the original mission of safety and practicality. By balancing innovation with original purpose, we can move from “hit-or-miss” to “precision hit”, ensuring that every PPN diagnosis is accurate, safe, and right for the patient.

## Funding

This work was supported by the 10.13039/501100001809National Natural Science Foundation of China (No. 82270116) and Pudong New Area Health and Family Planning Commission (No. 2024-PWXZ-05).

## Declaration of competing interest

All authors declare that they have no known competing financial interests or personal relationships that could have appeared to influence the work reported in this paper.

## CRediT authorship contribution statement

**Ximing Liao:** Writing – review & editing, Writing – original draft, Conceptualization. **Qiang Li:** Investigation, Conceptualization. **Ko-pen Wang:** Supervision, Investigation, Conceptualization.

## Appendix Supplementary materials

Image, application 1
