# Supplementary material for: From “shooting in the dark” to “precision hit”: Technological evolution, synergistic strategies, and future paths for peripheral pulmonary nodule diagnosis
Source: Chin Med J Pulm Crit Care Med. 2026 Jun 6;4(2):95–8. doi: 10.1016/j.pccm.2026.03.001 (PMC13323528; doi:10.1016/j.pccm.2026.03.001)
Supplement: Supplementary file 1 [file mmc1.docx]

**
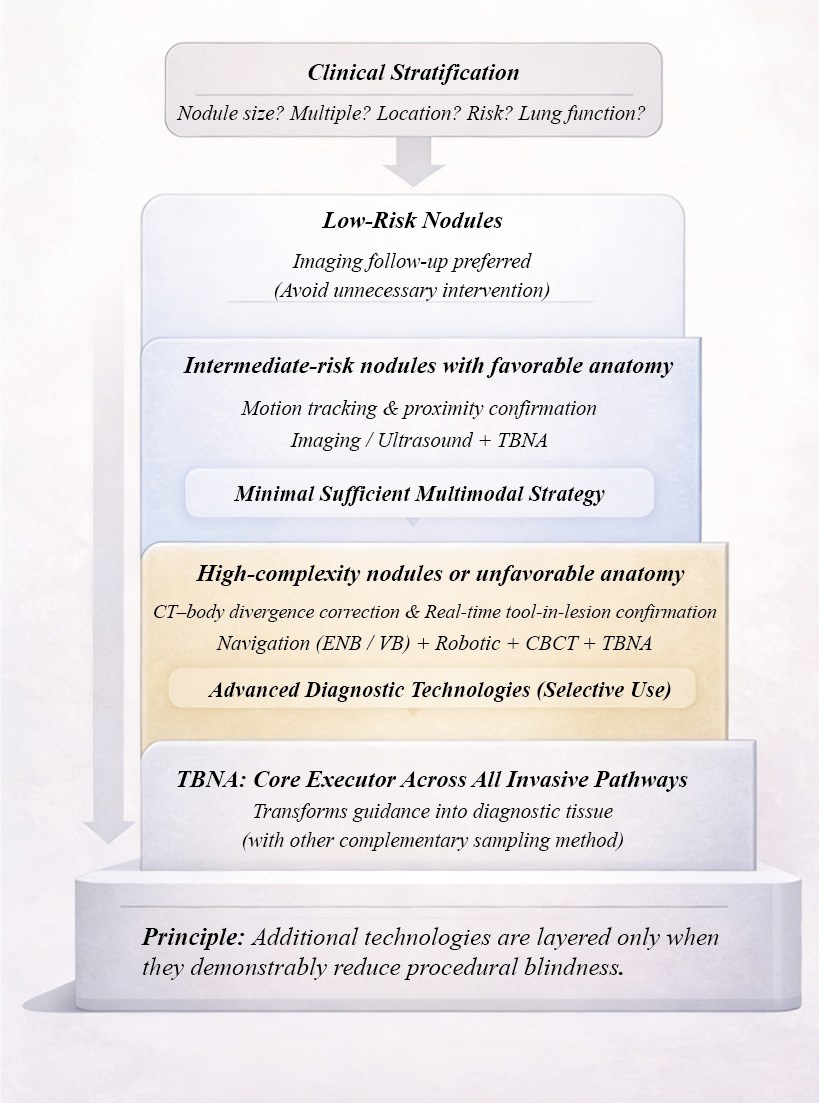
**

**Supplementary Fig. 1.** A TBNA-anchored, decision-oriented diagnostic ladder for peripheral pulmonary nodules. This schematic illustrates a stepwise diagnostic strategy based on clinical complexity rather than technological maximalism. Low-risk nodules are managed with surveillance to avoid unnecessary intervention. Standard PPN cases are addressed using minimal sufficient multimodal combinations, such as fluoroscopy and RP-EBUS, to mitigate respiratory motion and localization uncertainty. Advanced navigation or robotic platforms with CBCT are selectively reserved for high-complexity cases to correct CT to body divergence. Across all invasive pathways, TBNA and complementary biopsy techniques serve as the core executor that converts guidance into diagnostic tissue. But additional technologies are layered only when they demonstrably reduce procedural blindness. CBCT: Cone-beam computed tomography; ENB: Electromagnetic navigation bronchoscopy; PPNs: Peripheral pulmonary nodules; RP-EBUS: Radial probe endobronchial ultrasound; TBNA: Transbronchial needle aspiration; VB: Virtual bronchoscopy.
